# APJ regulates the balance between self-renewal and differentiation of vascular endothelial stem cells

**DOI:** 10.1186/s41232-025-00389-y

**Published:** 2025-08-04

**Authors:** Man Wang, Fitriana Nur Rahmawati, Wenting Li, Zeynep Bal, Faya Nuralda Sitompul, Fumitaka Muramatsu, Weizhen Jia, Nobuyuki Takakura

**Affiliations:** 1 Department of Signal Transduction, Research Institute for Microbial Diseases, The University of Osaka, 3-1 Yamada-Oka, Suita, Osaka 565-0871 Japan; 2 World Premier Institute Immunology Frontier Research Center, The University of Osaka, 3-1 Yamada-Oka, Suita, Osaka 565-0871 Japan; 3 Integrated Frontier Research for Medical Science Division, Institute for Open and Transdisciplinary Research Initiatives (OTRI), The University of Osaka, 3-1 Yamada-Oka, Suita, Osaka 565-0871 Japan; 4 Center for Infectious Disease Education and Research, 3-1 Yamada-Oka, Suita, Osaka 565-0871 Japan

**Keywords:** Endothelial cells, Endothelial cell differentiation, Endothelial stem cells, Vascular repair

## Abstract

**Background:**

CD157 marks a population of tissue-resident vascular endothelial stem cells (VESCs) in mice known for their critical role in homeostatic endothelial cell (EC) turnover and the rapid response to vascular damage in the liver by regeneration. Nevertheless, the mechanism underlying the maintenance and differentiation of postnatal VESCs under both physiological and pathological conditions remains unclear.

**Methods:**

APJ knockout (KO) mice were utilized to explore the role of apelin/APJ signaling in VESC functionality. Flow cytometry, colony-forming unit assays, and in vitro differentiation experiments were conducted to characterize VESC populations. Partial hepatectomy (PHx) was performed to assess vascular regeneration.

**Results:**

APJ deficiency led to an accumulation of VESCs in the liver of adult mice, which displayed enhanced colony-forming capacity but delayed differentiation into mature ECs. APJ KO mice exhibited impaired vascular regeneration following PHx, linked to compromised VESC differentiation. Transcriptomic analysis revealed upregulation of transcription factors EGR1 and EGR2 and downregulation of Ccnd1 in APJ KO VESCs, implicating disrupted cell cycle regulation. Additionally, APJ deletion reduced collagen IV levels, weakening the basement membrane and contributing to the maintenance of VESCs in an undifferentiated state.

**Conclusion:**

APJ signaling is critical for balancing VESC self-renewal and differentiation. APJ deficiency disrupts this balance, leading to impaired vascular regeneration in the liver due to delayed VESC differentiation. This defect is associated with altered transcriptional regulation, favoring a proliferative, undifferentiated state and extracellular matrix changes that weaken structural integrity. These findings highlight the apelin/APJ pathway as a potential therapeutic target to enhance vascular regeneration in regenerative medicine.

**Supplementary Information:**

The online version contains supplementary material available at 10.1186/s41232-025-00389-y.

## Introduction

The formation of the embryonic vasculature is a highly orchestrated process, beginning with vasculogenesis followed by angiogenesis. Vasculogenesis involves the de novo formation of primitive vessel plexuses by differentiated endothelial cells (ECs) from mesodermal precursor cells. After this initial stage, angiogenesis occurs as new blood vessels are generated from in-situ predecessors, thereby expanding and refining the vascular network [1]. In adults, blood vessel ECs typically remain quiescent. However, for tissue repair or under pathological conditions, such as tumor growth or cardiovascular injury, resident ECs from neighboring vessels will proliferate to form new blood vessels. As a specific EC subpopulation, extensive studies of tissue-resident vascular endothelial stem cells (VESCs) have demonstrated their enhanced clonal proliferative and vessel-forming capacity compared to mature ECs. In response to tissue injury, they can expand and differentiate into mature cells and finally regenerate blood vessels [2–4].

CD157, a glycoprotein expressed on the surface of ECs, has been identified as a marker for VESCs [4, 5]. Our previous studies have shown that CD157-positive (CD157^+^) ECs are present in various organs and tissues, including but not limited to the lung, heart, limb muscles, skin, retina, and brain. Such cells exhibit greater generative potential and higher colony-forming ability from single cells compared to their CD157-negative (CD157^−^) EC counterparts. Several animal studies have shown that CD157^+^ VESCs actively participate in the repair process of many organs or tissues and can be a potential treatment regimen. For example, in the liver, VESCs are capable of regenerating entire vascular structures following injury and supporting large vessels in a healthy liver for over 1 year. Additionally, transplanted CD157^+^CD200^+^ VESC-derived ECs have been shown to secrete coagulation factor VIII and effectively rescue a bleeding phenotype in hemophiliac models [4]. In a chronic cerebral hypoperfusion mouse model, the transplantation of brain-derived CD157^+^ ECs induced angiogenesis with significantly fewer white matter lesions and reduced brain dysfunction compared to CD157^−^ ECs [6]. Our previous single-cell temporal transcriptomic study of ECs from fetal, neonatal, and adult mice disclosed the developmental trajectory of fetal CD157^−^CD200^+^ VESC-like cells to early postnatal CD157^+^CD200^+^ VESCs and then to mature ECs [7]. Nevertheless, it remains unclear how postnatal VESCs maintain self-renewal or differentiate into mature ECs.

The apelin receptor (APJ) is a G protein-coupled receptor with seven transmembrane domains for binding with bioactive apelin ligands expressed in the membranes of ECs [8]. The apelin/APJ signaling pathway is widely distributed across the cardiovascular system during the lifespan of mice. In mouse embryos, APJ deficiency results in partial embryonic lethality, characterized by markedly deformed vasculature in the yolk sac and embryo, poorly looped hearts with aberrant right ventricular formation, and defective atrioventricular cushion development [9]. During embryogenesis, arterial ECs produce apelin, which induces chemotaxis of venous ECs and promotes the production of secreted Frizzled-related protein 1 (sFRP1) by APJ^+^ venous ECs, thereby regulating arterial–venous alignment for thermoregulation [10]. Moreover, APJ signaling is essential for the generation of hematopoietic cells. Deletion of APJ disrupts hematopoietic stem and progenitor cell (HSPC) production in embryonic hemangioblasts, while activation of the APJ pathway hinders the generation of long-term reconstituting HSPCs, favoring myeloid differentiation [11]. In adult mouse bone marrow, apelin is expressed in a unique EC precursor subpopulation crucial for bone marrow regeneration after injury. In other words, these apelin-positive endothelial niche cells regulate hematopoiesis and vascular regeneration following myeloablative injury [12].

In this study, we explored the role of apelin/APJ signaling in regulating the maintenance, differentiation, and regenerative potential of VESCs, both in vitro and in vivo, using an APJ knockout (KO) mouse model. Moreover, we explored the potential underlying mechanisms that may drive these processes.

## Methods

### Animals

C57BL/6 J mice were obtained from Japan SLC Inc. (Shizuoka, Japan). The generation of APJ KO mice was described previously [10, 13]. To assess breeding performance and viability, homozygous intercrosses (APJ^–/–^ × APJ^–/–^) were conducted. A total of 46 litters were analyzed, yielding 1–6 pups per litter (median 3–4), which was lower than the typical litter size of 6–8 pups observed in wild-type (WT) C57BL/6 J mice under the same conditions. These findings are consistent with the previously reported partial embryonic lethality observed in APJ-deficient mice [9]. The mice were kept under specific pathogen-free conditions in a controlled environment in an animal experimentation facility at Osaka University under 12-h light/dark cycles. All animal experiments followed the guidelines of the Osaka University Committee for Animal and Recombinant DNA Experiments.

### Murine model of partial hepatectomy

A partial hepatectomy mouse model was adapted from a method described previously [14]. Briefly, female mice aged 8–12 weeks were anesthetized using a combination of three agents: 4.0 mg/kg midazolam (Sandoz, Holzkirchen, Germany), 0.3 mg/kg medetomidine (Domitor, Nippon Zenyaku Kogyo, Fukushima, Japan), and 5.0 mg/kg butorphanol (Vetorphale, Meiji Seika Pharma, Tokyo, Japan). A midline abdominal incision, approximately 3 cm in length, was made through the skin and muscle, and the peritoneal cavity was opened to allow access to the liver. The median and left lateral lobes of the liver were sequentially resected. This was accomplished by encircling the base of each lobe with a 4–0 silk suture and placing the knot as close to the base as possible. The tied lobe was then excised just above the suture using microsurgical curved scissors. After resection, the abdomen was closed, and the skin surrounding the incision was wiped with betadine. To reverse the anesthesia, 0.3 mg/kg of atipamezole (Antisedan, Nippon Zenyaku Kogyo Co., Ltd.) was administered. The mice were then placed on a warming pad to recover from anesthesia.

### EC isolation and flow cytometric analysis

Primary cell isolation was conducted following previously reported protocols with slight modifications [4, 15]. Briefly, liver and mammary tissues were harvested and rinsed with 4% fetal bovine serum (FBS; HyClone, Thermo Scientific, Yokohama, Japan)/phosphate-buffered saline (PBS). The tissues were minced with sharp scissors for 15 min to facilitate cell dissociation. A single-cell suspension was then prepared using a digestion mixture containing dispase (Wako, Osaka, Japan, cat. no. 034–22363), collagenase (Worthington Biochemical, Lakewood, NJ, USA; cat. no. LS004176), collagenase II (Gibco, Waltham MA, USA; cat. no. 17105–041), and DNase (Roche, Basel, Switzerland; cat. no. 11284932001). Specifically, 7 mL of pre-warmed enzyme solution was added to each 15-mL conical tube containing tissues, and the tubes were then incubated in a water bath at 37 °C for 5 min. The tubes were mechanically shaken at 250 rpm for 20 min at 37 °C. After digestion, the solution was filtered through 40-µm cell strainers (Falcon, Fisher Scientific, Waltham, MA, USA, cat. no. 352340), and red blood cells were removed using ACK lysing buffer (Lonza, Basel, Switzerland, cat. no. 10-548E). For cell sorting, the following antibodies were used at a 1:200 dilution: anti-mouse CD16/CD32 (Mouse BD Fc Block™; BD PharMingen, Franklin Lakes, NJ, USA; clone #2.4G2), anti-CD31 (clone MEC13.3; BD Biosciences, Franklin Lakes, NJ, USA), anti-CD45 (clone 30-F11; BD Biosciences), anti-CD157 (clone BP3; BioLegend, San Diego, CA, USA), anti-CD200 (clone OX90; BioLegend), and anti-protein C receptor (Procr; clone eBio1560; eBioscience, San Diego, CA, USA).

For cell-cycle analysis, cell suspensions were prepared and surface marker staining was performed according to the above standard protocols. Cells were fixed with 200 µL of 4% paraformaldehyde (PFA)/PBS on ice for 20 min, followed by two washes with 4% FBS/PBS. The cells were resuspended in 1 mL of freshly prepared 4′,6-diamidino-2-phenylindole (DAPI) working solution (10 µg/mL DAPI in 0.1% Triton X-100 in PBS) and incubated in the dark for 30 min at room temperature.

All cell analyses and sorting were performed using a FACS system (BD FACSAria™ Cell sorter, BD LSRFortessa™ Cell AnalyzerX-20). Flow cytometry data were analyzed using FlowJo software (Version X, Ashland, OR, USA).

### Cell culture and endothelial colony formation assay

Freshly isolated ECs were cultured on fibronectin (Sigma-Aldrich, St Louis, MI, USA; F1141)-coated 96-well plates in HuMedia-EG2 (Kurabo, Osaka, Japan) containing 20% FBS and 10 ng/mL VEGF165 (PeproTech, Rocky Hill, NJ, USA) at 37 °C in a humidified 5% CO_2_ atmosphere. Fresh medium was supplied every 2 days.

For EC colony–forming assays, freshly isolated (1 × 10^3^) ECs were plated onto 24-well plates and co-cultured with OP9 cells (RIKEN Cell Bank, Tsukuba, Japan). Cultures were maintained in RPMI-1640 medium (Sigma–Aldrich Japan, Tokyo, Japan) supplemented with 10% FBS (Sigma–Aldrich), 1% penicillin/streptomycin (Sigma–Aldrich), and 10^−5^ mol/L 2-mercaptoethanol (Thermo Fisher Scientific, Waltham, MA, USA) with the addition of VEGF165 at 10 ng/mL every 3 days. To examine the downstream signaling of apelin/APJ, (Pyr^1^)-Apelin-13 (10 ng/mL; BACHEM, Japan K.K., Tokyo, Japan; cat # 217,082–60-5) was added on day 12. For pathway inhibition, LY294002 (PI3K inhibitor; 1 μM; Selleck Houston, TX, USA; cat # S1105) or Y-27632 (ROCK inhibitor; 500 nM; Selleck; cat# S6390) was added 6 h before apelin stimulation.

### Quantitative PCR

Total mRNA was extracted from ECs using an RNeasy Plus Mini Kit (Qiagen, Hilden, Germany; cat. no./ID 74134) following the manufacturer’s protocol. The isolated RNA was then reverse transcribed into cDNA utilizing a PrimeScript RT reagent Kit (Takara Bio, Shiga, Japan; #RR037A). Quantitative real-time PCR (qPCR) was conducted using a LightCycler 96 System (Roche, Basel, Switzerland). Data were processed using a 2^−∆∆*Ct*^ method and normalized against the internal reference gene, *GAPDH*. The specific primers used are listed in Supplementary Table S1.

### Immunohistochemical analysis

Cultured colony-forming ECs were initially rinsed with PBS and then fixed for 15 min at room temperature using 4% PFA/PBS. Following fixation, the cells were washed with PBS and blocked for 2 h at 4 °C in a blocking buffer composed of PBS plus 0.05% Tween-20 (PBST) supplemented with 5% normal goat serum, 1% bovine serum albumin, and 0.2% skim milk. Overnight incubation at 4 °C with rat anti-mouse CD31 antibody (clone MEC13.3 BioLegend) was performed. On the second day, after washing, the cells were then treated with a biotin-labeled goat anti-rat IgG secondary antibody (Agilent Technologies, Santa Clara, CA, USA) for 2 h, followed by a 30-min incubation with ABC reagent (Vector Laboratories, Newark, CA, USA) at room temperature. For liver tissue samples, the samples were fixed overnight at 4 °C in 4% PFA/PBS. The tissues were cryoprotected by immersion in 15% and 30% sucrose solutions until fully saturated, embedded in Tissue-Tek O.C.T. compound (Sakura Finetek, Tokyo, Japan), and sectioned into 50-µm slices. After fixation with 4% PFA and blocking, tissue sections were incubated overnight at 4 °C with primary antibodies, including rat anti-mouse CD31 (clone MEC13.3 BioLegend), hamster anti-mouse CD31 (clone 2H8, Merck Millipore, Burlington, MA, USA), mouse anti-mouse CD157 PE (clone BP3; BioLegend), rabbit anti-mouse APJ (clone 5H5L9; Invitrogen; Waltham, MA, USA), rabbit anti-Mo/Rt Ki-67 (clone SolA15 eBioscience), and rabbit anti-mouse COL4 (ab6586, Abcam, Cambridge, UK). After washing, the sections were incubated with appropriate secondary antibodies, such as goat anti-rat Alexa Fluor 488 (Thermo Fisher), goat anti-hamster Alexa Fluor 488 (Thermo Fisher), and goat anti-rabbit Alexa Fluor 647 (Thermo Fisher). The slides were mounted with Dako Fluorescent Mounting Medium (Dako, Carpinteria, CA, USA). Images were captured using a Canon EOS Kiss X7 camera (Canon, Ota City, Japan), a Leica TCS SP5 confocal microscope (Leica Microsystems, Wetzlar, Germany), and an Olympus FV3000 confocal microscope (Olympus, Tokyo, Japan). Image analysis was conducted using ImageJ (NIH) and Fuji (NIH) software.

#### Statistical analysis

All statistical analyses were carried out using GraphPad Prism v9.0 (GraphPad Software, Inc., CA, USA). The results are reported as mean ± standard deviation (SD). Two-tailed unpaired Student’s *t*-tests were used to evaluate the population mean difference. One-way analysis of variance (ANOVA) was applied for single-factor comparisons among multiple groups, and two-way ANOVA was employed for comparisons involving multiple factors. Hypothesis tests with *P*-values of less than 0.05 were considered statistically significant. The *P*-values are reported as ^∗^*P* < 0*.*05, ^∗∗^*P* < 0*.*01, and ^∗∗∗^*P* < 0*.*001.

## Results

### APJ deficiency increases VESC population in adult mice

**Fig. 1:**
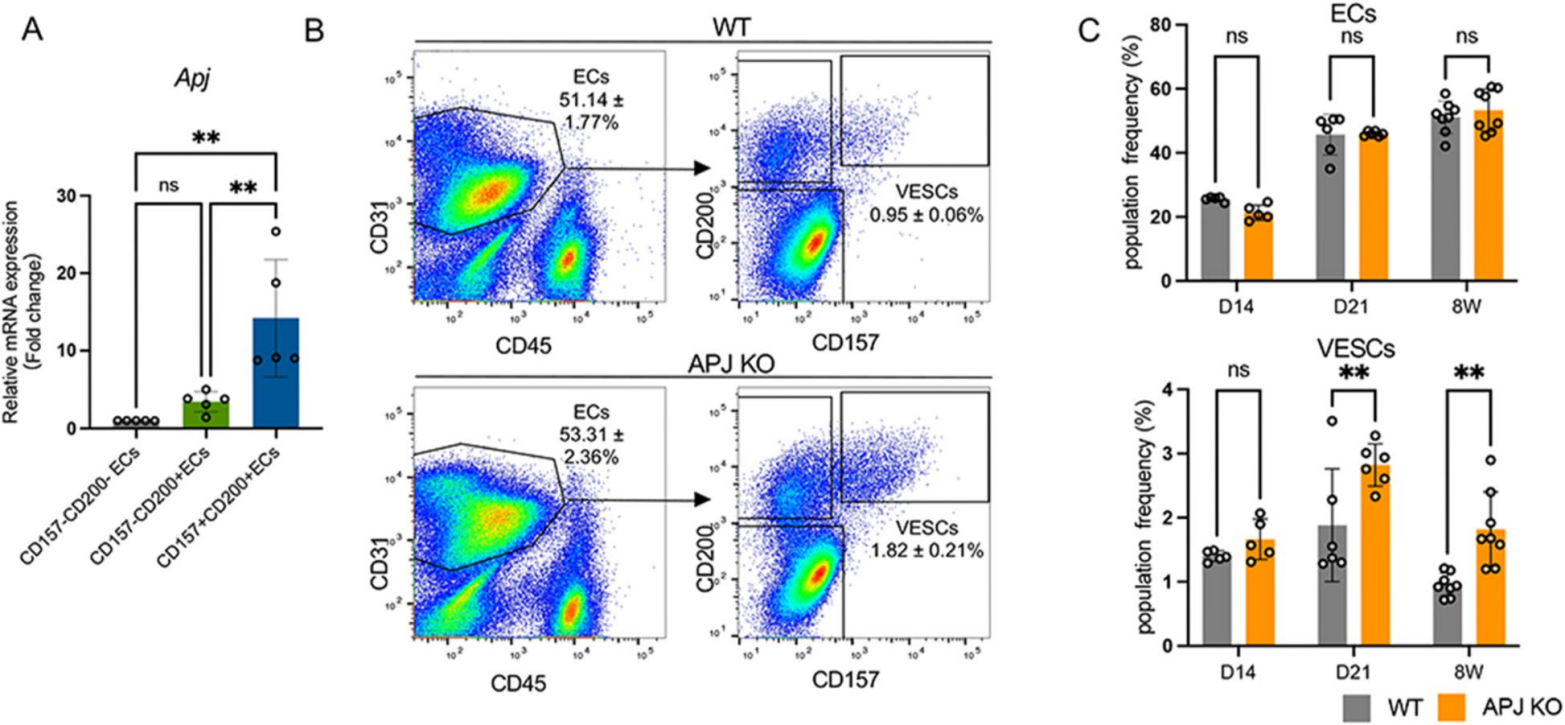
APJ deficiency increases the VESC population in adult mice.** A** Relative mRNA expression levels of APJ in CD157^−^CD200^−^, CD157^−^CD200^+^, and CD157^+^CD200^+^ liver ECs were quantified using qPCR (*n* = 5). **B** Representative FACS plots from WT and APJ KO mice. The left-hand panels show PI^−^-gated populations after doublet exclusion, and the right-hand panels show the further analysis of CD31^+^CD45^−^ ECs for CD157 expression. Statistical data are presented as mean ± SEM and are indicated on the respective graphs within the figure. **C** Quantification of total ECs and CD157^+^CD200^+^ VESCs in the liver at different time points (day 14, day 21, and 8 weeks; *n* = 5–8). Statistical significance was assessed by one-way ANOVA in **A** and two-way ANOVA in **C** **P < 0.01. ns, not significant

The APJ is highly enriched in ECs of the embryonic liver vasculature, but its expression declines significantly in the adult stage [16]. In our analysis, using previously published single-cell RNA-sequencing data [17], we observed that APJ^+^ cells were predominantly present within the first week of birth, after which these cells declined dramatically by postnatal day 21 (D21) and, in the end, became a tiny cell population (Fig. S1A). Additionally, our immunofluorescence staining at postnatal days 1, 3, 7, 21, and 56 confirmed this trend in APJ expression over time (Fig. S1B). Further gene expression analysis revealed that APJ expression is significantly higher in a CD157^+^CD200^+^ VESC population compared to CD157^−^CD200^−^ terminally differentiated ECs or CD157^−^CD200^+^ progenitor EC populations (Fig. 1A). CD157^+^ VESCs mainly localized in the portal vein of adult mice, which was the physical source for vasculogenesis [4]. Hence, a functional association between APJ signaling and the homeostasis of VESCs in adult mice is postulated. To assess the role of APJ in VESCs, we utilized an APJ KO mouse model based on the observation that the overall liver EC population size remained unaffected by APJ deletion. However, the VESC component within the population grew significantly after D21 and was maintained in adult (8-weeks-old; 8 W) APJ KO mice (Fig. 1B, C). To validate this finding, we extended our research to VESC populations in other organs, i.e., in the mammary gland where the VESC population was suggested to express Procr [3]. Consistent with prior results in the liver, APJ deficiency caused a marked increase in the proportion of VESCs during adulthood rather than a change in the general EC population size (Fig. S2).

### APJ deficiency increases VESC colony formation but hinders VESC differentiation in vitro

**Fig. 2:**
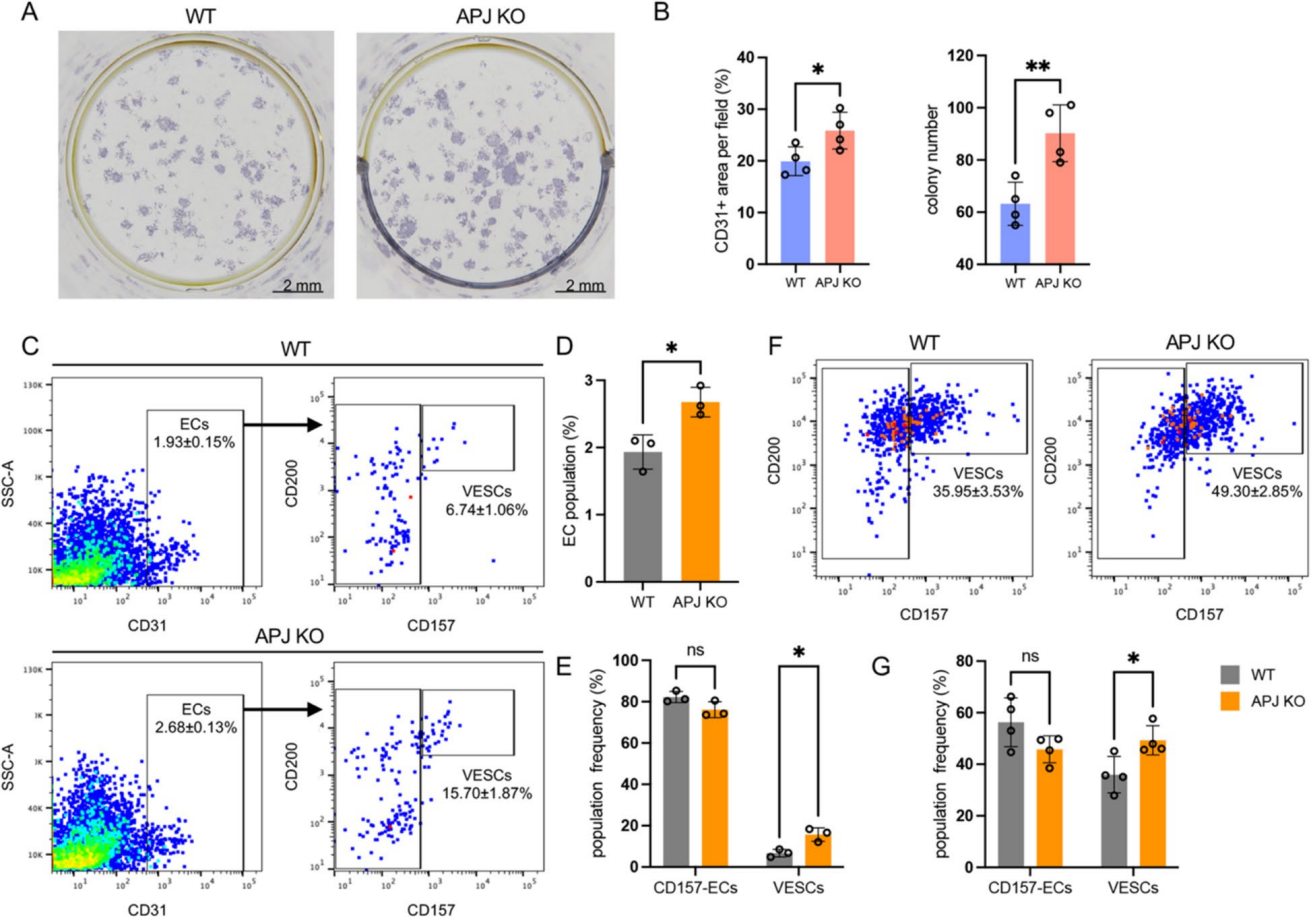
APJ deficiency increases VESC colony formation but hinders VESC differentiation in vitro.** A** Representative images of colonies formed by VESCs isolated from the livers of WT and APJ KO mice stained with anti-CD31 antibody. **B** Quantification of CD31-positive colony areas and numbers (*n* = 4). **C** Representative FACS plots showing colonies formed by VESCs cultured on OP9 cells from WT and APJ KO mice. **D** and **E** Quantification of total EC population and CD157^+^CD200^+^ EC population frequency from colonies shown in (**C**) (*n* = 3). **F** Representative FACS plots of VESCs cultured on fibronectin-coated plates from WT and APJ KO mice. **G** Quantification of the VESC population from cultures shown in **F** (*n* = 4). Statistical data are presented as Mean ± SEM and are indicated on the respective graphs within the figure. Statistical significance was assessed with two-tailed unpaired Student’s *t*-tests in **B** and **D** and two-way ANOVA in **E** and **G** *P < 0.05 or **P < 0.01. ns, not significant

Our previous studies revealed that CD157^+^ VESCs co-cultured with OP9 stromal cells showed enhanced colony formation compared to CD157^− ^ECs [4]. In this study, VESCs were isolated from APJ KO and WT adult mice and co-cultured with OP9 stromal cells to exclude individual environmental factors of APJ deficiency. We then compared the effect of APJ deficiency on CD157^+^ VESCs by assessing their colony-forming efficiency and EC amplification. CD157^+^ VESCs isolated from APJ KO mice exhibited both a notable increase in colony number and size compared to those from WT mice (Fig. 2A, B). This indicated that APJ deficiency promoted the maintenance of stemness in VESCs at a more immature state, allowing for a greater proliferative capacity. To further explore this possibility, we harvested ECs from this culture and conducted a flow cytometric analysis. Consistent with our prior immunohistochemistry findings, VESCs isolated from APJ KO mice exhibited a larger EC population and a higher proportion of CD157^+^ VESCs compared to WT mice, with no difference observed in CD157^−^ ECs (Fig. 2C–E). This suggests that APJ deficiency preserves the proliferative capacity of VESCs while delaying their differentiation.

To specifically investigate the effect of APJ deficiency on VESC self-renewal and maintenance in an undifferentiated state, VESCs were cultured on fibronectin-coated dishes, creating a simplified environment devoid of external differentiation cues. Flow cytometry analysis confirmed that CD157 positivity in ECs was significantly maintained in VESCs from APJ KO compared to WT mice, alongside a slight, non-significant reduction in mature ECs, and this suggests that APJ deficiency hinders the differentiation of CD157^+^ VESCs (Fig. 2F, G).

### APJ deficiency impairs EC regeneration after partial hepatectomy

**Fig. 3:**
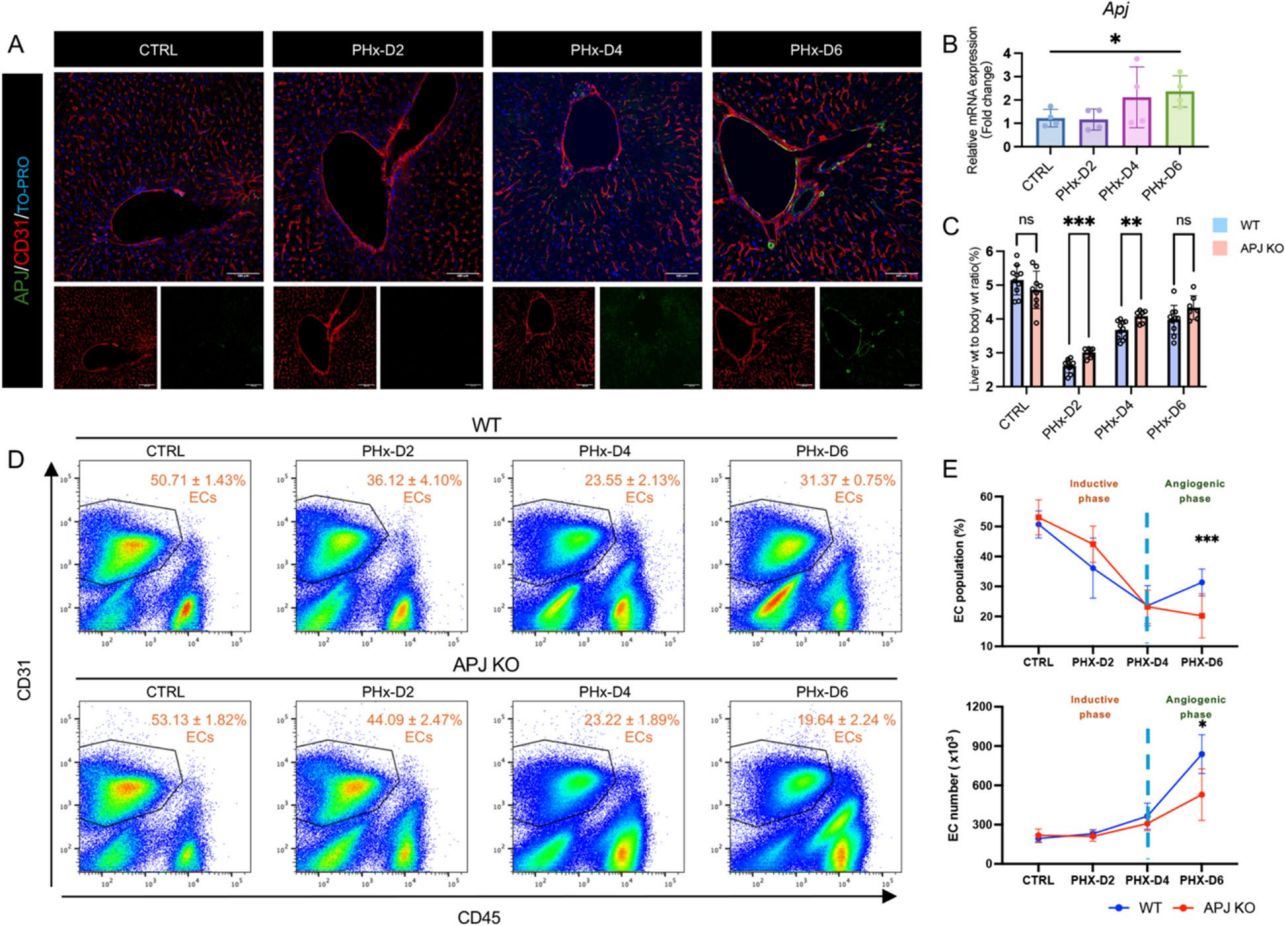
APJ deficiency impairs EC regeneration after PHx.** A** Immunofluorescence staining showing APJ (green), CD31 (red) expression, and nuclei (blue) in liver sections from WT mice at indicated days post-PHx. **B** Relative mRNA expression levels of APJ in liver ECs from WT mice were quantified by qPCR at different time points post-PHx (CTRL, day 2, 4, and 6; *n* = 4). **C** Liver weight to body weight ratio at different time points post-PHx (*n* = 9–11). **D** Representative FACS plots displaying dynamic changes in the EC population frequency at specified time points post-PHx. Statistical data are presented as mean ± SEM and are indicated on the respective graphs within the figure. **E** The upper line graph shows statistical quantification of EC population frequency changes over time post-PHx; the lower line graph shows total EC counts in the liver, with the CTRL group representing the right and caudate lobes, and the post-PHx groups representing the remaining liver lobes (right and caudate) after PHx (*n* = 6–10). Statistical significance was assessed with one-way ANOVA in **B** and two-tailed unpaired Student’s *t*-tests in **C** and **E** *P < 0.05, **P < 0.01, or ***P < 0.001. ns, not significant

CD157^+^ VESCs can expand and regenerate vascular structures following liver injury. In cases where the liver endothelium remains intact, only in-situ ECs participate in liver vascular regeneration [18]. To examine the role of APJ in promoting in-situ vascular regeneration, we utilized a partial hepatectomy (PHx) model that involves the removal of approximately 70% of the liver. The liver regeneration occurs sequentially in two phases: an early inductive phase and a later angiogenic phase [19]. Our experiment showed that both the transcribed RNA and translated protein of APJ significantly increased in expression by day 6 post-PHx during liver vessel regeneration (Fig. 3A, B). A seemingly controversial finding was that the APJ KO mice displayed accelerated liver recovery, evidenced grossly by body-weight ratios during the early inductive phase (Fig. 3C). This enhanced recovery may be attributed to increased hepatocyte growth factor (HGF) secretion by ECs in APJ KO mice, which further promoted hepatocyte proliferation (Fig. S3A and 3B). These findings aligned with previous studies showing that blocking APJ can augment early liver regeneration [20]. Nevertheless, flow cytometry and immunofluorescence analyses revealed delayed EC regeneration in APJ KO mice in the late angiogenic stage, particularly on day 6 after surgery (Fig. 3D, E; Fig. S4). This delay suggests that APJ deficiency may promote early liver regeneration and significantly impair the regeneration of blood vessels, thereby affecting long-term tissue recovery and function.

### APJ deficiency prevents VESC differentiation during vascular regeneration

**Fig. 4:**
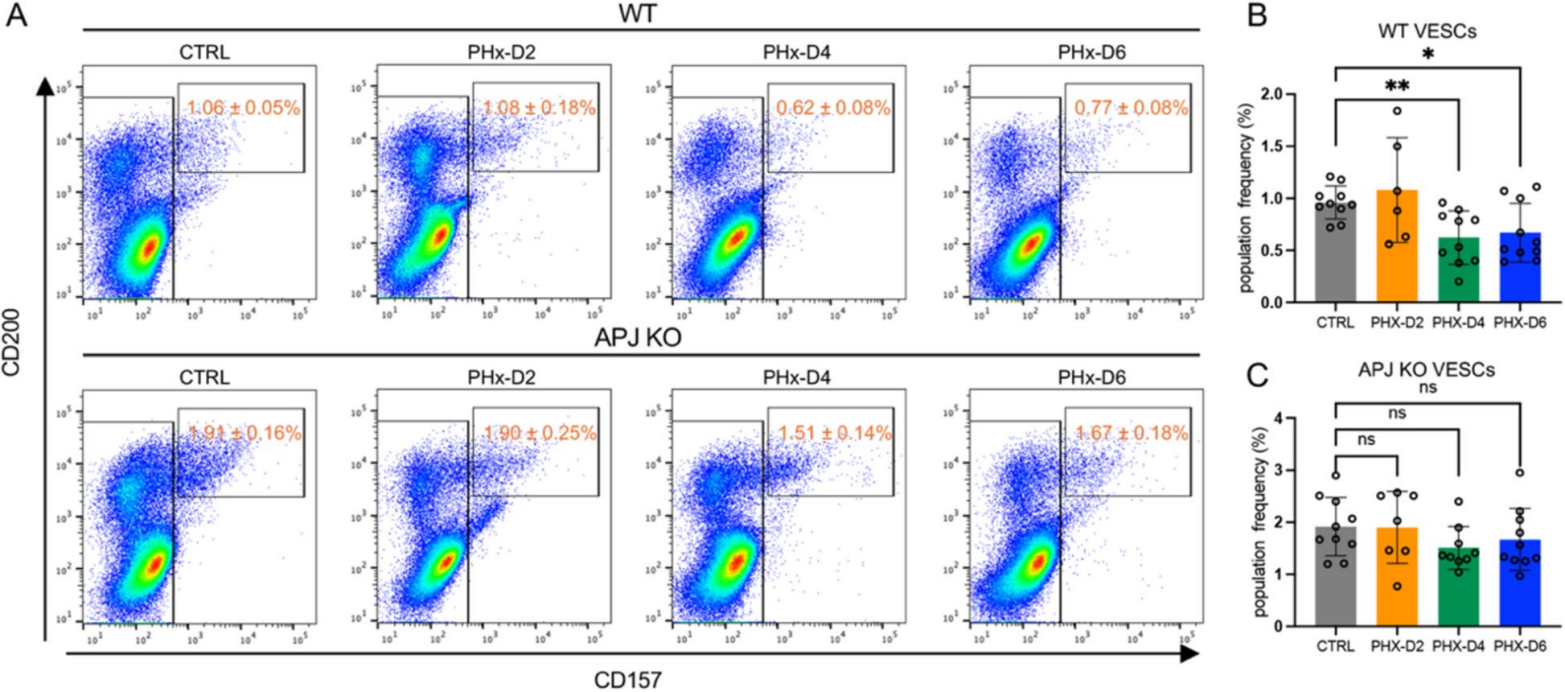
APJ deficiency prevents VESC differentiation during vascular regeneration.** A** Representative FACS plots showing VESCs among CD31^+^CD45^−^ ECs at various time points post-PHx, illustrating changes in the EC population. Statistical data are presented as mean ± SEM and are indicated on the respective graphs within the figure. **B** and **C** Statistical quantification of VESCs within the total EC population as represented in the FACS plots in **A** (*n* = 6–10). Statistical significance was assessed with one-way ANOVA. *P < 0.05, **P < 0.01

To further investigate the cause of impaired vessel regeneration in APJ KO mice, we analyzed EC subpopulations during the regenerative process. In WT mice, the VESC population declined significantly from day 4 post-PHx, coinciding with the start of the angiogenic phase, suggesting that VESCs actively differentiate into mature ECs to rebuild the vascular network in pathological conditions (Fig. 4A, B). Possibly half of the CD157^+^ VESCs undergo symmetric cell division, i.e., one CD157^+^ VESC gives rise to two CD157^−^ ECs after cell division. In contrast, in APJ KO mice, the VESC population size remained constant, suggesting CD157^+^ECs undergo asymmetric cell division, i.e., one CD157^+^ VESC provides both a CD157^+^ VESC and a CD157^−^ EC, which may explain this discrepancy (Fig. 4A, C). This delay in vascular regeneration, along with the maintenance of VESCs in an undifferentiated state, strongly suggests that APJ regulates proper VESC differentiation and promotes EC function during liver regeneration. Thus, the increased VESC count in APJ KO mice likely reflects impaired differentiation rather than functional cell expansion, leading to compromised vascular repair.

### Altered cell-cycle progression and transcription factor expression in VESCs of APJ KO mice

**Fig. 5:**
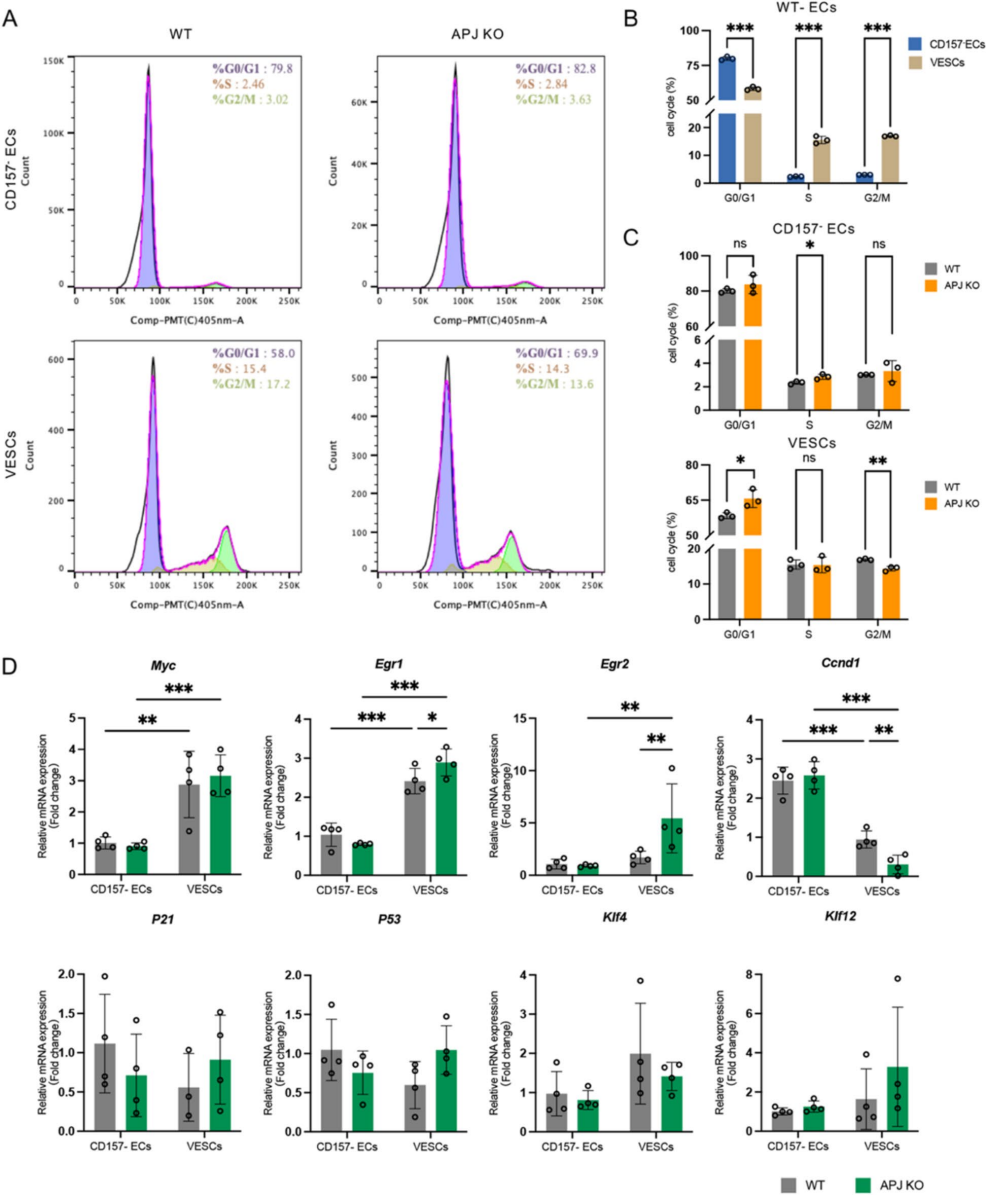
Altered cell-cycle progression and transcription factor expression in VESCs of APJ KO mice.** A** Representative flow cytometric histograms showing cell-cycle distribution (G1/G0, S, and G2/M phases) of CD157^−^ECs and VESCs from WT and APJ KO mice. **B** Percentages of cells in each phase (G1/G0, S, and G2/M) for CD157^−^ECs and VESCs in WT mice (*n* = 3). **C** Comparison of cell-cycle phase percentages for CD157^−^ECs and VESCs between WT and APJ KO mice (*n* = 3). WT data are reused here to enable a direct comparison with the KO group. **D** Relative mRNA expression levels of cell-cycle–related transcription factors were measured in CD157.^−^ECs and VESCs from WT and APJ KO mice (*n* = 4). Statistical significance was assessed using two-tailed unpaired Student’s *t*-tests in **B** and **C** for comparisons within each phase and two-way ANOVA in **D**. *P < 0.05, **P < 0.01, ***P < 0.001

To investigate the effect of APJ on the cell cycle and differentiation of VESCs, we examined cell-cycle dynamics in liver VESCs (Fig. 5A). Compared to CD157^−^ ECs, VESCs displayed a higher proportion of cells in the S and G2/M phases, reflecting their active proliferative state (Fig. 5B). However, in APJ KO VESCs, we observed a shift in the cell cycle, with an increase in the G1 phase and a corresponding decrease in the G2 phase, suggesting a disruption in normal cell-cycle progression (Fig. 5C). Common transcription factors that are associated with regulating cell differentiation or cell-cycle progression were measured. In both WT and APJ KO mice, VESCs exhibited increased expression of *Myc* and *Egr1* and downregulated *Ccnd1* transcription factors compared to mature ECs, highlighting the proliferative capacity of VESCs and a delicate balance between proliferation and differentiation. Compared to WT mice, VESCs in APJ KO mice exhibited even greater levels of *Egr1* and *Egr2* and a lower level of *Ccnd1* (Fig. 5D). Changes in these transcriptional factors, including unknown factors that may disrupt the balance between self-renewal and differentiation in APJ KO VESCs, intrinsically favor self-renewal and inhibit differentiation.

### Extracellular matrix remodeling in VESCs of APJ KO mice

**Fig. 6:**
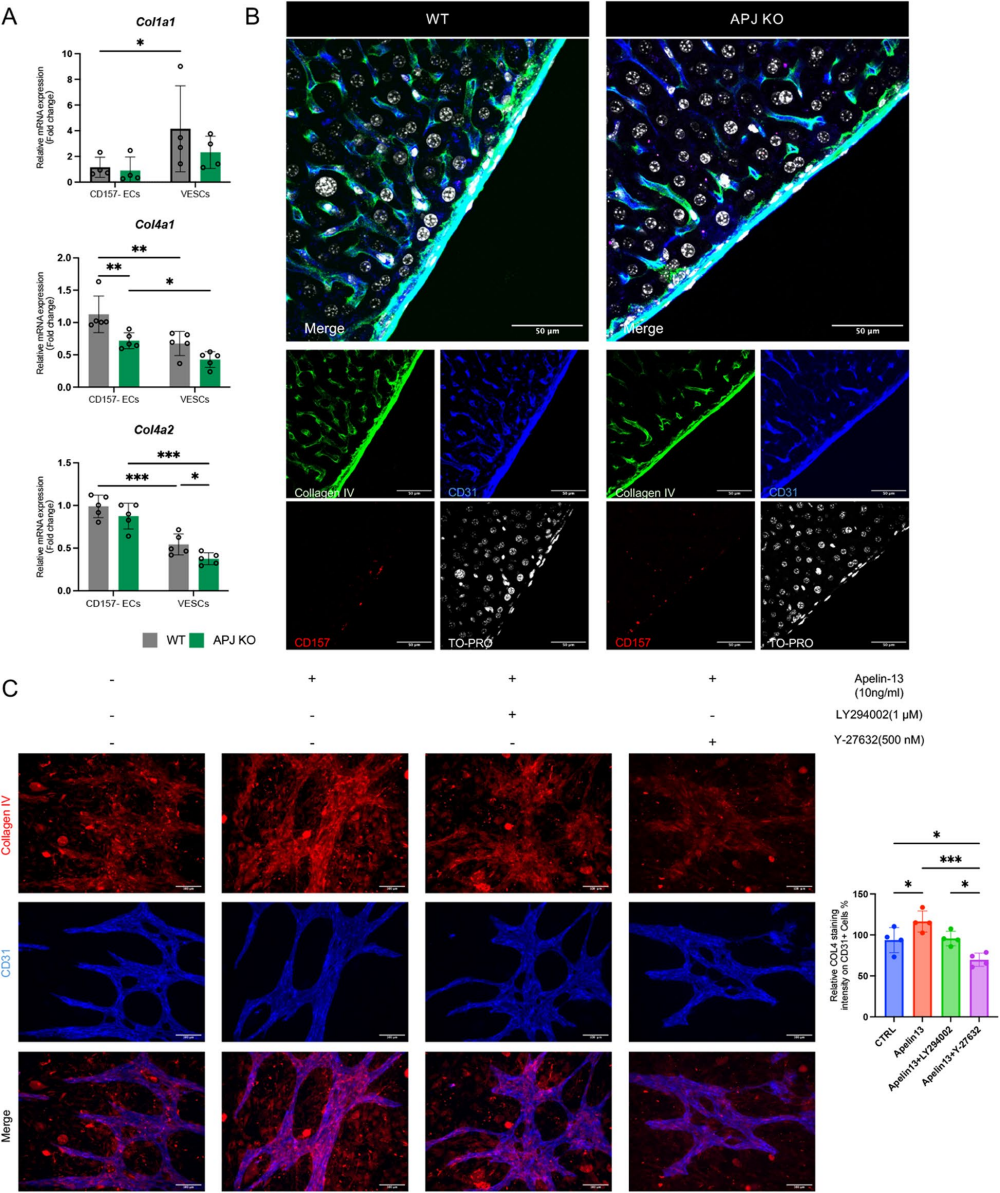
ECM remodeling in VESCs of APJ KO mice. **A** Relative mRNA expression levels of ECM-related genes, including *Col1a1*, *Col4a1*, and *Col4a2* (*n* = 4), in CD157^−^ECs and VESCs harvested from WT and APJ KO mice. **B** Immunofluorescence analysis of COL4 in liver tissues from WT and APJ KO mice. CD31 (blue), CD157 (red), COL4 (green), and DAPI (gray) are shown. **C** Representative immunofluorescence images of COL4 (red) and CD31 (blue) in VESCs co-cultured with OP9 stromal cells. Apelin was added on day 12, and pharmacological inhibitors targeting either the PI3K (LY294002) or RhoA/ROCK (Y-27632) pathways were applied 6 h before apelin stimulation. Cells were fixed and stained on day 14. The right panel shows quantification of COL4 fluorescence intensity within CD31⁺ endothelial regions (mean ± SD, *n* = 4 per group). Statistical significance was assessed using two-way ANOVA. *P < 0.05, **P < 0.01, ***P < 0.001

Cell-cycle regulation by the alteration of transcriptional factors is one mechanism to induce the accumulation of VESCs in APJ KO mice; however, we also investigated extrinsic factors affecting VESC self-renewal or differentiation. Given the well-established role of the extracellular matrix (ECM) in guiding stem cell differentiation, we hypothesized that the impaired differentiation of VESCs in APJ KO mice could be linked to changes in ECM composition [21, 22]. The mRNA of genes related to the ECM was measured: *Col4a1* and *Col4a2*, linked to the intactness of the basement membrane, were significantly downregulated in VESCs of APJ KO mice compared to those of the WT (Fig. 6A). Immunofluorescence analysis further confirmed these results by revealing a marked reduction of collagen IV (COL4) deposition around the vasculature in the liver of APJ KO mice (Fig. 6B). COL4 is essential for maintaining the integrity of the stem cell niche and for providing the signals necessary for proper differentiation. These results suggest that the reduction of COL4 may weaken the basement membrane and disrupt the signaling required for VESC differentiation and impaired vascular regeneration. To further investigate the signaling pathways regulating COL4 expression, we treated VESC–OP9 co-cultures with apelin from day 12 and applied PI3K or RhoA/ROCK inhibitors 6 h prior to apelin treatment. Apelin stimulation enhanced COL4 expression, whereas RhoA/ROCK inhibition, unlike PI3K inhibition, suppressed COL4 deposition even in the presence of apelin (Fig. 6C). These results indicate that APJ regulates COL4 expression at least in part through the RhoA/ROCK pathway and that disruption of this signaling axis may contribute to the impaired function of VESCs under APJ-deficient conditions.

## Discussion

Vascular endothelial stem cells reside in exclusive locations and play a critical role in maintaining vascular homeostasis. Their number and function are tightly regulated to ensure the appropriate homeostatic turnover of ECs and to enable rapid regenerative responses to vascular damage or disease. This regulation involves a delicate balance between quiescence, self-renewal, expansion, and differentiation [23, 24]. However, despite the recognized importance of VESCs, the mechanisms governing these processes in postnatal VESCs remain poorly understood. In the present study, a global APJ KO mouse model was employed. Results showed that VESCs highly express APJ, and APJ deficiency led to the significant accumulation of VESCs in adult mouse livers. To minimize confounding factors from other cell types, VESCs were isolated and examined in vitro, enabling a controlled assessment of APJ deficiency. Notably, APJ-deficient VESCs exhibited delayed differentiation in both in vitro cultures and in vivo during vascular regeneration. In vitro, this delayed differentiation appeared to enhanced the colony formation of immature ECs. However, it caused impaired vascular regeneration, particularly following PHx in vivo.

Stem cell fate decisions are tightly linked to intrinsic cell-cycle dynamics, with different division modes—symmetric self-renewal, asymmetric division, and symmetric differentiation—often associated with distinct cell-cycle states [25]. The expansion of VESCs in APJ-deficient mice raises the possibility that APJ may influence VESC fate through cell-cycle regulation. To address this, we examined cell-cycle progression in VESCs.

Our findings showed that liver VESCs, both in WT and APJ KO mice, had a higher proportion of cells in the S and G2/M phases compared to terminally differentiated ECs, reflecting their proliferative potential and active involvement in EC renewal [26–28]. However, in APJ-deficient mice, this balance was disrupted: VESCs showed a shift toward the G0/G1 phase and a decrease in the G2/M phase, implying delayed cell-cycle progression. Transcriptional profiling revealed a concomitant increase in the expression of *Egr1* and *Egr2* transcription factors, along with a marked reduction in *Ccnd1* transcription factor levels. These changes suggest a disruption in normal cell-cycle dynamics that may favor the maintenance of stemness over progression toward differentiation. Interestingly, EGR1 is known to play context-dependent roles in stem cell regulation. In hematopoietic stem cells, EGR1 supports quiescence and long-term maintenance, whereas in regenerative contexts, it can promote differentiation [29, 30]. We previously identified EGR1 as a positive regulator of CD157 expression in ECs, and its upregulation in APJ-deficient VESCs may contribute to sustaining an undifferentiated state [31]. The concurrent downregulation of *Ccnd1* further supports the notion that APJ loss delays cell-cycle progression, hindering VESC differentiation [32].

Moreover, previous studies have indicated that APJ signaling can directly modulate cell-cycle regulators. Granulosa cells derived from patients with Turner syndrome, which exhibit impaired APJ ligand signaling, display reduced expression of CTPS2 and a shift toward the G0/G1 arrest. This altered profile could be partially rescued by APJ ligands or by activating the downstream AKT pathway, highlighting a mechanistic link between APJ and cell-cycle progression [33]. Given that AKT is known to regulate EGR1, EGR2, and Ccnd1 expression, the transcriptional changes observed in APJ-deficient VESCs are likely mediated through a disrupted APJ–AKT axis.

Across stem cell systems, APJ exhibits stage- and lineage-specific roles. In hematopoiesis, it promotes the early emergence of HSPCs from mesodermal progenitors but must be downregulated for long-term HSC maintenance [11]. In the nervous system, APJ signaling promotes oligodendrocyte differentiation, with APJ deletion resulting in hypomyelination and APJ activation enhancing remyelination [34]. These examples reinforce a model in which APJ plays distinct, context-dependent roles in regulating the balance between stem cell maintenance and differentiation across various tissues.

Beyond regulating cell-cycle progression intrinsically, our results suggest that APJ also influences stem cell fate extrinsically by modulating the ECM microenvironment.

In mature tissues, *COL4A1* and *COL4A2* are the principal α-chain genes encoding collagen IV, a key structural component of the vascular basement membrane. They are crucial for maintaining endothelial integrity, vessel stability, and selective barrier function [35, 36]. Their expression fluctuates, i.e., decreasing during angiogenic sprouting and increasing during EC maturation [37]. Clinically, mutations in *COL4A* genes can result in fragile vasculature, leading to conditions such as intracerebral hemorrhages and microbleeds, underscoring the critical role of COL4 in vascular stability [38]. Beyond structural support, the ECM, particularly COL4, provides biochemical cues that regulate stem cell maintenance, self-renewal, and differentiation [39]. Recent advances in engineered hydrogel systems that mimic ECM microenvironments have further demonstrated that both ECM composition and mechanical properties critically influence endothelial lineage specification from stem and progenitor cells [40]. In our study, undifferentiated VESCs exhibited lower COL4 expression, consistent with a state less dependent on basement membrane attachment, allowing their contribution to EC renewal and vascular repair. In APJ KO adult mice, COL4 expression was markedly reduced in liver ECs, which aligns with previous reports that APJ deficiency increases vascular permeability [41, 42]. Reduced COL4 expression also likely impairs VESC differentiation and hinders vascular regeneration. This notion is further supported by findings from planarian regeneration models, where COL4 is essential for the transition from stem cell proliferation to differentiation. During regeneration, COL4 accumulated in the blastema and was required for the transition from stem cell proliferation to differentiation. Loss of COL4 leads to persistent neoblast hyperproliferation, impaired progenitor formation, and defective tissue regeneration. Mechanistically, COL4 signals through the collagen receptor DDR-1 in niche neurons to suppress the EGF-like ligand NRG-7, thereby limiting excessive self-renewal. These findings underscore a conserved role of COL4 in promoting stem cell differentiation [21]. Accordingly, reduced COL4 expression in APJ-deficient mice may similarly compromise the endothelial stem cell niche, leading to impaired VESC differentiation and delayed vascular regeneration.

Although not directly addressed in the present study, potential compensatory responses to APJ deficiency, such as activation of alternative endothelial progenitor pools or GPCR-mediated signaling pathways, may also contribute to maintaining vascular homeostasis and warrant future investigation [43].

Accumulating evidence highlights the important role of apelin/APJ signaling in vascular regeneration across various pathological conditions. Apelin and APJ are upregulated in ECs of multiple tissues in response to systemic hypoxia, which promotes the formation of enlarged, non-leaky blood vessels and enhances vascular stability during the recovery process [44]. Moreover, apelin/APJ signaling also marks endothelial stem-like cells that contribute critically to vascular regeneration in the lung and bone marrow following injury. In models of acute lung injury, apelin⁺ endothelial stem-like cells give rise to proliferative APJ⁺ progenitors that drive microvascular regeneration [45]. Similarly, a rare population of apelin⁺ ECs in the bone marrow is essential for reestablishing vascular integrity and supporting hematopoietic recovery post-irradiation [12]. Our findings extend this regenerative paradigm to the liver, revealing a dual role for APJ in hepatic regeneration. While APJ deficiency transiently accelerates parenchymal recovery, it impairs vascular repair by disrupting VESC differentiation. These results underscore the conserved and multifaceted functions of apelin/APJ signaling in orchestrating endothelial stability, stem cell activation, and tissue regeneration. Importantly, they raise the possibility that targeting APJ signaling at specific regenerative windows may offer a promising therapeutic strategy to enhance endothelial repair without compromising early parenchymal recovery. Future studies investigating molecular mediators downstream of APJ will be essential to fully elucidate its therapeutic potential in regenerative medicine.

## Supplementary Information

Supplementary Material 1.

## Data Availability

The datasets used and/or analyzed during the current study are available from the corresponding author on reasonable request.

## Abbreviations

ANOVA: Analysis of variance
APJ: Apelin receptor
COL4: Collagen IV
ECs: Endothelial cells
ECM: Extracellular matrix
HSPC: Hematopoietic stem and progenitor cell
HGF: Hepatocyte growth factor
KO: Knockout
PHx: Partial hepatectomy
Procr: Protein C receptor
sFRP1: Secreted Frizzled-related protein 1
