# Supplementary material for: APJ regulates the balance between self-renewal and differentiation of vascular endothelial stem cells
Source: Inflamm Regen. 2025 Aug 4;45:25. doi: 10.1186/s41232-025-00389-y (PMC12323185; doi:10.1186/s41232-025-00389-y)
Supplement: Supplementary file 1 — Supplementary Material 1. [file 41232_2025_389_MOESM1_ESM.docx]

**Supplementary information**

**Figure S1. APJ^+^ Cells Are Predominantly Present During the First Week Post-Birth and Decline by Postnatal Day 21. A**, ScRNA-seq data was used to analyze APJ expression in liver ECs at various postnatal time points (Days 1, 3, 7, 21, and 56). The line graph illustrates the trend in the proportion of APJ^+^ ECs relative to the total EC population over time. **B**, Representative immunofluorescence staining of APJ in liver tissues at various time points (Days 1, 3, 7, 21, and 56). CD31 (red), APJ (green), and DAPI (blue) are shown. The areas indicated by the white boxes are magnified in the images below right.

**
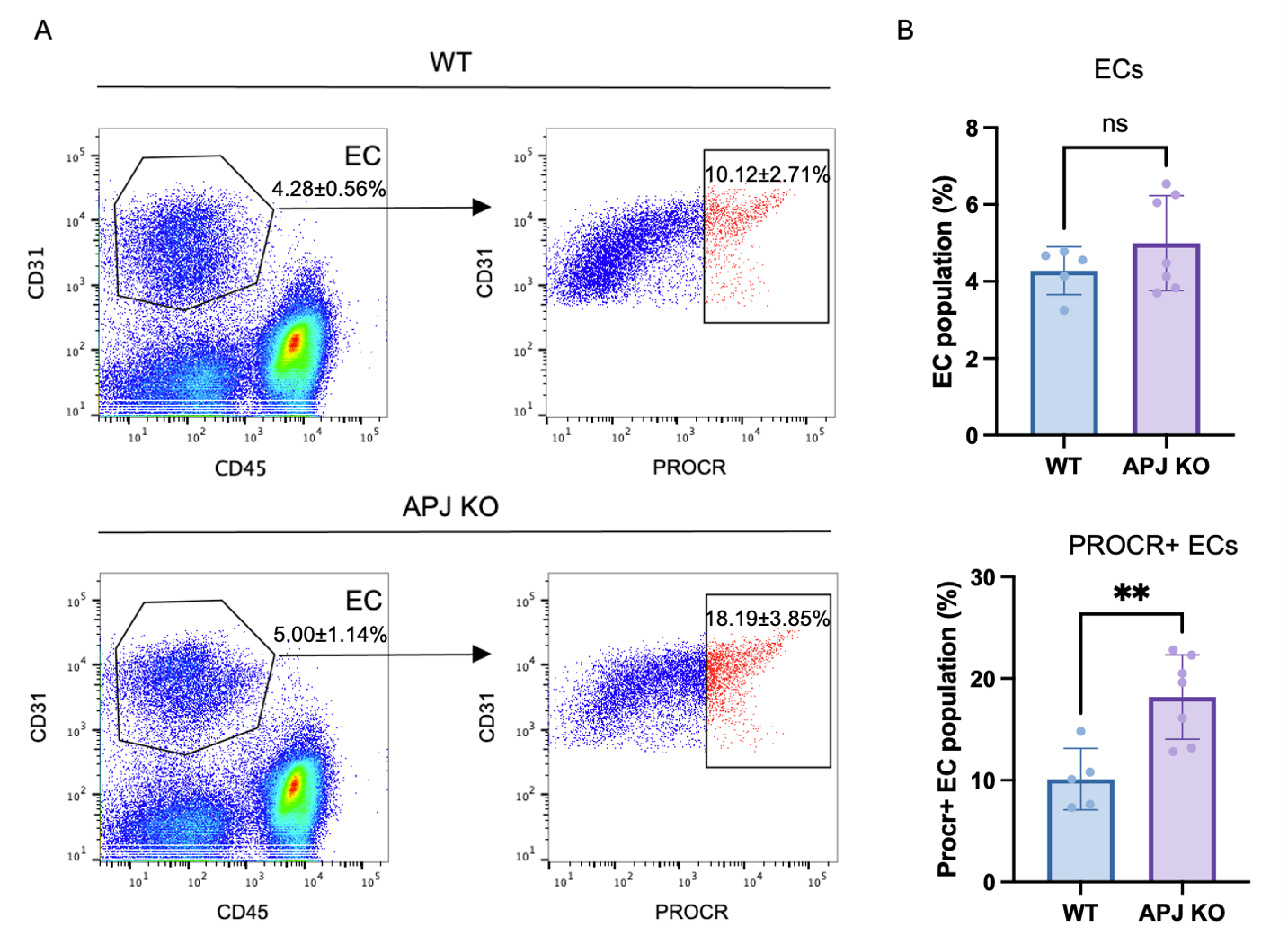
Figure S2** **APJ deficiency increases VESC population in adult mice mammary glands. A**, Representative FACS plots from WT and APJ KO mice mammary glands. The left panels show PI^−^-gated populations after doublet exclusion; the right-hand panels show further analysis of CD31+CD45− ECs) for Procr expression. Statistical data are presented as Mean ± SEM and are indicated on the respective graphs within the figure. **B**, The quantification and statistical significance of total ECs and procr ^+^ ECs in the mammary gland were using two-tailed unpaired Student’s *t*-tests. ***P* < 0.01(n=5, 7).

**
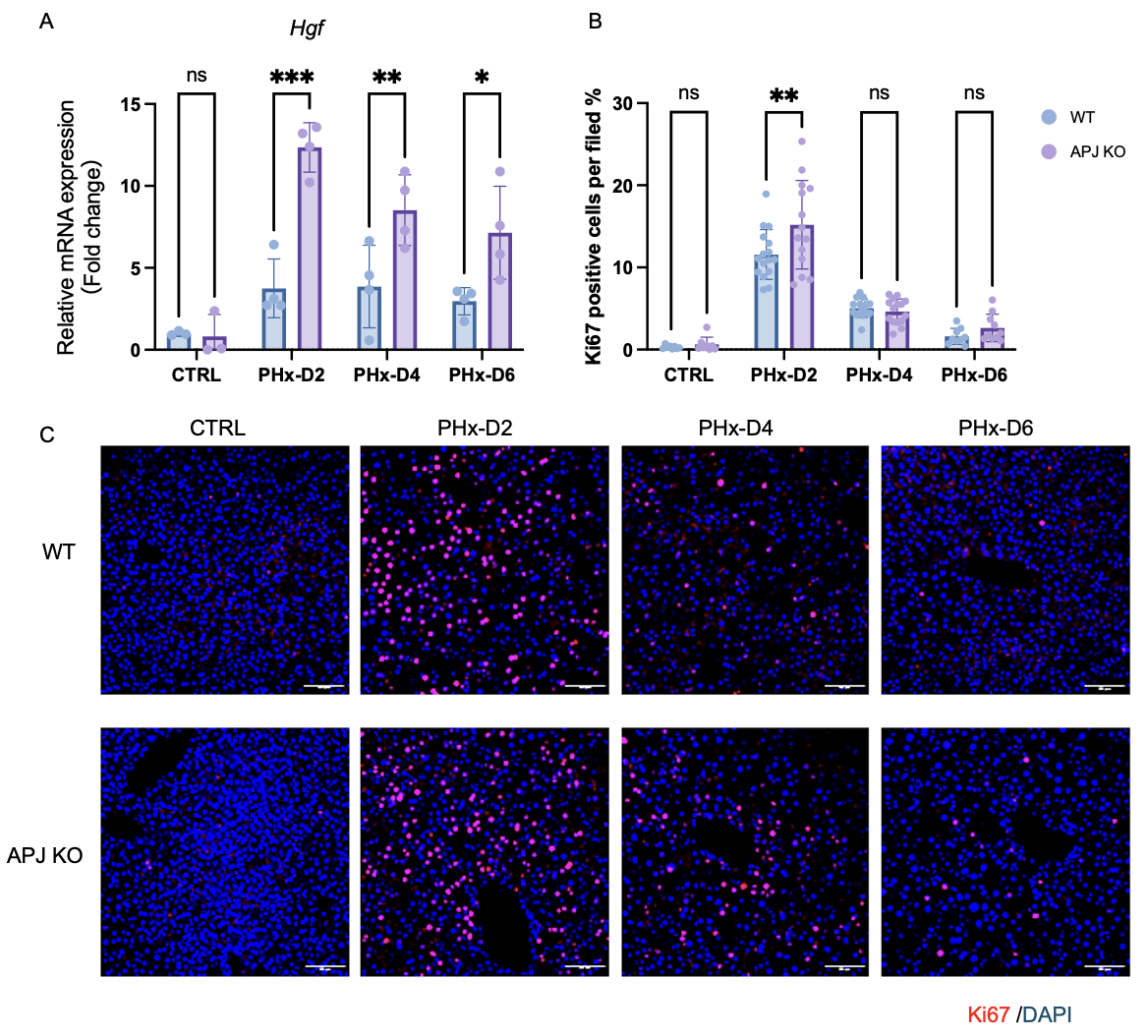
**

**Figure S3. A**, Relative mRNA expression levels of HGF in liver ECs from WT and APJ KO mice were quantified at different time points post-PHx; (CTRL, Days 2, 4, and 6) using qPCR (n = 3–4). **B**, Statistical quantification of the ratio of Ki67-positive cells based on randomly selected regions from each group (n=3–4, per group), as shown in (**C**). **C**, Representative immunofluorescence staining of Ki67 (red) in the liver of WT and APJ KO mice at different time points post-PHx (CTRL, Days 2, 4, and 6), with nuclei stained by DAPI (blue). Statistical significance was assessed using two-way ANOVA. **P* < 0.05, ***P* < 0.01, ****P* < 0.001 or ns, not significant.

**Figure S4. A**, Representative immunofluorescence staining of CD31(red) and LYVE-1 in the liver of WT and APJ KO mice at different time points post-PHx (CTRL, Days 2, 4, and 6). **B**, The areas of CD31⁺ and LYVE-1⁺ structures were quantified as percentages of the total tissue area. Data are presented as mean ± SD (n = 5–7 mice per group). Statistical significance was assessed using two-tailed unpaired Student’s t-tests. *p < 0.05.

**Supplementary Table S1** Primers used in the present study

| Gene | Forward (5’>3’) | Reverse (5’>3’) |
| --- | --- | --- |
| *Apj* | CCACTGTGGGCCACTTATACC | CAGCCTTAGCCGAGCATTG |
| *Col4a1* | CTGGCACAAAAGGGACGAG | ACGTGGCCGAGAATTTCACC |
| *Col4a2* | CCCGGATCTGTACAAGGGTG | TGATGCCTTCCTCGCCTTTT |
| *Myc* | AGTGCTGCATGAGGAGACAC | GGGTTTGCCTCTTCTCCACA |
| *Egr1* | AACCGGCCCAGCAAGACACC | TGCAGATTCGACACTGGAAG |
| *Egr2* | CTTCAGCCGAAGTGACCACC | GCTCTTCCGTTCCTTCTGCC |
| *Klf4* | CTATGCAGGCTGTGGCAAAACC | TTGCGGTAGTGCCTGGTCAGTT |
| *Klf12* | ATGATGACCTGCCAAATGTGACCT | CTGGATACCGGGGATGGATGT |
| *P53* | AAAGGATGCCCATGCTACAG | TATGGCGGGAAGTAGACTGG |
| *P21* | TAGGGGAATTGGAGTCAGGC | AGAGTGCAAGACAGCGACAA |
| *Ccnd1* | TCAAGTGTGACCCGGACTG | ATGTCCACATCTCGCACGTC |
| *Gapdh* | AACTTTGGCATTGTGGAAGG | GGATGCAGGGATGATGTTCT |
